# Rapid and simple analysis of short and long sequencing reads using Duesselpore^TM^

**DOI:** 10.3389/fgene.2022.931996

**Published:** 2022-08-11

**Authors:** Christian Vogeley, Thach Nguyen, Selina Woeste, Jean Krutmann, Thomas Haarmann-Stemmann, Andrea Rossi

**Affiliations:** IUF-Leibniz Research Institute for Environmental Medicine, Düsseldorf, Germany

**Keywords:** Oxford nanopore sequencing, next-generation sequencing, RNA-seq analyses, NGS data analysis, Oxford Nanopore MinION device

## Abstract

Transcriptome analysis experiments enable researchers to gain extensive insights into the molecular mechanisms underlying cell physiology and disease. Oxford Nanopore Technologies (ONT) has recently been developed as a fast, miniaturized, portable, and cost-effective alternative to next-generation sequencing (NGS). However, RNA-Seq data analysis software that exploits ONT portability and allows scientists to easily analyze ONT data everywhere without bioinformatics expertise is not widely available. We developed Duesselpore^TM^, an easy-to-follow deep sequencing workflow that runs as a local webserver and allows the analysis of ONT data everywhere without requiring additional bioinformatics tools or internet connection. Duesselpore^TM^ output includes differentially expressed genes and further downstream analyses, such as variance heatmap, disease and gene ontology plots, gene concept network plots, and exports customized pathways for different cellular processes. We validated Duesselpore^TM^ by analyzing the transcriptomic changes induced by PCB126, a dioxin-like PCB, and a potent aryl hydrocarbon receptor (AhR) agonist in human HaCaT keratinocytes, a well-characterized model system. Duesselpore^TM^ was specifically developed to analyze ONT data, but we also implemented NGS data analysis. Duesselpore^TM^ is compatible with Linux, Microsoft, and Mac operating systems and allows convenient, reliable, and cost-effective analysis of ONT and NGS data.

## Introduction

In the past decade, RNA-sequencing (RNA-Seq) has become the leading method for analyzing whole-genome transcriptomes (Ashley, 2016). RNA-Seq is used in modern medicine for diagnosis, prognosis, and therapeutic selection in the fields of infectious disease, fetal monitoring, and cancer (Ku and Roukos, 2014; Ashley, 2016). The ability to sequence DNA and RNA quickly and inexpensively is essential to scientific research (Wheeler et al., 2008). This necessity has prompted the development of many sequencing techniques beyond the original Sanger sequencing (Shendure and Ji, 2008). The rise of next-generation sequencing (NGS) has considerably improved the output of data generated by Sanger sequencing (first-generation sequencing) (Buermans et al., 2017). NGS approaches have become widespread in research and diagnostic laboratories and have greatly increased our knowledge of many genetic disorders (Schuster, 2007; Shendure and Ji, 2008; Rivas et al., 2011; Jamuar et al., 2014; König et al., 2015). Nevertheless, these technologies remain expensive, laborious, time-consuming, and affected by the limitations of short-read sequencing. As some kind of a new generation, new sequencing methods were established, which aim to sequence long DNA or RNA molecules (Slatko et al., 2018), including Oxford Nanopore Technologies (ONT).

ONT sequencing is based on transmembrane proteins (nanopores) embedded into a lipid membrane and measures changes in electric current across these pores. These changes are caused by nanosized molecules, such as DNA or RNA (or even amino acids), as they occupy a volume that interferes with the ion flow and can be recorded by a semiconductor-based electronic detection system and afterward translated into sequences (Hornblower et al., 2007; Branton et al., 2008). In the last few years, ONT sequencing has proven to be a fast and cost-effective alternative to other sequencing techniques. Even if the read quality does not reach the high standard of other techniques, it offers other advantages such as fast and easy library preparation, real-time sequencing, PCR-free, direct RNA, and ultra-long read sequencing (Liu et al., 2019; Xu and Seki, 2019). The latter is especially useful for improving *de novo* assembly, mapping certainty, transcript isoform identification, and detection of structural variants (Jain et al., 2018).

Additionally, some ONT sequencers are small, granting portability, and enabling sequencing experiments outside the laboratory. Nevertheless, sequencing analysis produces large raw datasets that require bioinformatics skills, familiarity with statistics, and a Linux-based environment to decode this information. Performing multiple RNA-Seq analysis workflows requires the handling of dozens of dependent libraries and third-party software, and individual data preparation. It would be ideal to circumvent this issue by integrating multiple workflows without requiring additional data preparation steps.

Available software allows robust analysis of those data, but they usually require an internet connection and basic bioinformatic skills, lack a friendly user interface (Ewels et al., 2020; Williams, 2022), or are mainly limited to basic analysis (Epi2me).

Furthermore, scientists might be unwilling or even not allowed (e.g., human datasets) to upload their unpublished data to an online platform to employ open-access web services. We believe that a client-based computational approach implemented in Javascript would represent an alternative solution that shifts the computation workload to the client-side. We recently deployed a light computational web server solution to analyze genome editing experiments from ONT data (Nguyen et al., 2022). However, RNA-Seq workflows are more complicated and have not been translated or compiled into lightweight Javascript-like software. Moreover, ONT offers mobile platforms that can work in environments where internet access is limited or absent.

**FIGURE 1:**
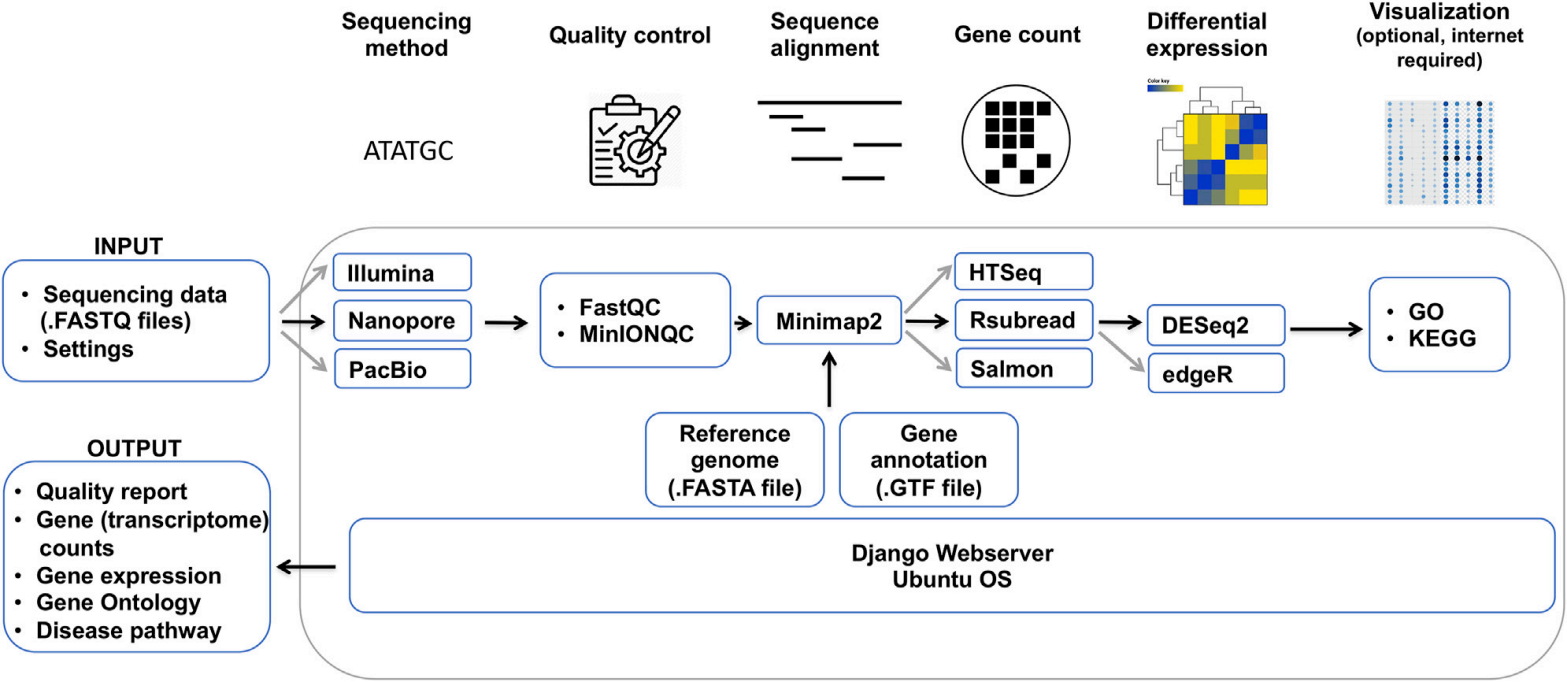
Bioinformatical data analysis pipeline of Duesselpore^TM^. Depicted are the required data, which need to be provided by the user, and the different steps of the data analysis, including quality control, sequence alignment, gene count, differential expression and visualization, and the output. Black arrows indicate the main workflow optimized for the analysis of ONT data, whereas grey arrows show the variations of the workflow that can be selected by the user.

In order to overcome these issues and help the community process ONT and other RNA sequencing datasets, we developed Duesselpore^TM^, a local web server that is particularly tailored to the portability of Nanopore sequencers. Instead of using a centralized system, we deploy an efficient local-based system that runs on a Docker container without the necessity of being connected to the internet. Running in a virtual environment has further advantages: portability, agility, scalability, and security, all empowered by Docker. Furthermore, the pipeline (Figure 1) runs in an isolated virtual environment and avoids the incompatibility of our workflow with the host’s software.

## Method and system description

### HaCaT cell culture and stimulation

HaCaT keratinocytes were cultivated at 37°C and 5% CO_2_ in DMEM medium (PAN Biotech, Aidenbach, Germany) with low glucose (1 g/L) supplemented with 10% FBS and 1% antibiotics/antimycotics (PAN Biotech). HaCaT keratinocytes were stimulated in full growth medium for 24 h with 1 µM PCB126 or were solvent treated using 0.1% DMSO.

### Library preparation

Total RNA was isolated using the GenUP Total RNA Kit (Biotechrabbit, Hennigsdorf, Germany). The High Sensitivity RNA ScreenTape System (Agilent Technologies, Santa Clara, CA) was used to assess the quality of isolated RNA. 50 ng of total RNA was reverse transcribed, and samples were barcoded with the PCR cDNA Barcoding Kit (SQK-PCB109, Oxford Nanopore Technologies, Oxford, United Kingdom). The quantity of amplified cDNA was then determined with the Qubit^TM^ 4 Fluorometer (Invitrogen, Carlsbad, CA), and the range of fragment size was assessed using the Agilent D1000 SreenTape assay (Agilent Technologies). The Flow Cell Priming Kit (EXP FLP002, Oxford Nanopore Technologies) was used to prime the flowcell (FLO-MIN106), and an equal amount of barcoded cDNA was loaded. Sequencing was carried out with a MinION (MN33710) using the MinKNOW software (v.21.02.1) over a period of 72 h.

### Base-calling

Raw FAST5 reads were base-called and demultiplexed using Guppy (v4.5.4 + 66c1a7753), reads with a quality score below seven were excluded using the following command:

guppy_basecaller --min_qscore 7 --trim_barcodes --barcode_kits "SQK-PCB109" --compress_fastq -i {input.fast5} -s {output.folder} -c dna_r9.4.1_450bps_hac.cfg --x auto --chunks_per_runner 128

The resulting FASTQ files of each sample were concatenated into one file using the following command:

On linux and mac OS terminal

$ cat /path/to/fastq/files/*.fastq > /your/new/location/output.fastq

On Windows command prompt (NOTE: path symbol is different):

$ type \path\to\fastq\files\*.fastq>\your\new\location\output.fastq

### Input data and run parameters

Duesselpore^TM^ validates the input by automatic format detection. Input FASTQ files are compressed and structured in each subdirectory named by experimental condition. Each subdirectory contains multiple biological replicates for each condition. The default parameters in the webserver form are optimized for analyzing long-read RNA-Seq data obtained from ONT. However, the users can change the settings based on their experimental setup and sequencing method of choice.

### Quality control

Our RNA-Seq workflow follows the standard data processing pipelines. First, we employ quality control with FastQC to quantify the sequence quality and export the quality into HTML files. Reads with a score below the predefined quality threshold (Q = 7) are filtered out.

### Align and assign reads to gene/transcriptome

Minimap2 (Li, 2018) was used to map the quality-filtered ONT reads to the reference genome/transcriptome. Aligned BAM files were processed by Rsubread/featureCounts (Liao et al., 2019), HTSeq/htseq-count (Anders et al., 2015), or Salmon (Patro et al., 2017) to generate the raw count of read genes or transcriptome. Although Salmon can quantify transcripts directly from the FASTQ reads (Liao et al., 2019), Minimap2 displays a higher assigned ratio for handling noisy ONT data (Williams, 2022).

### Differential expression analysis and gene ontology

The raw read count matrices are processed by the differential expression methods DESeq2 (Love et al., 2014) and edgeR/limma (Robinson et al., 2009; Ritchie et al., 2015). DESeq2 displays a high capability to process data with a small number of biological replicates, a common issue in NGS.

To analyze gene ontology, we integrated the gene ontology pipeline using Bioconductor. Results are clustered into various gene ontology pathways such as gene set enrichment analysis of gene ontology (gseGO), disease ontology, network of cancer genes (DOSE) (Yu et al., 2015), and pathview (Luo and Brouwer, 2013). Please note that whilst most of the analyses performed by Duesselpore^TM^ do not require an internet connection, gene ontology and disease pathway (optional function) will need an internet connection (see supplementary information).

### Web server and backend platform

Duesselpore^TM^ is implemented in Python 3.8 with Django 3.2 web framework, which has long-term support from the open-source community. Bioconda, Biopython, and R/Bioconductor run on the Ubuntu 18.04 system encapsulated in the Docker container as backend. Docker can be configured depending on the host PC. All software is free and open-source. The detailed system architecture and manual are described in the Supplementary document.

## Results

### Validation of Duesselpore^TM^

Duesselpore^TM^ is implemented in Python 3.8 with Django 3.2 web framework (Figure 1) and it encapsulates different pipelines including quality control, sequence alignment, gene count, differential expression, and visualization (Figure 1).

**FIGURE 2:**
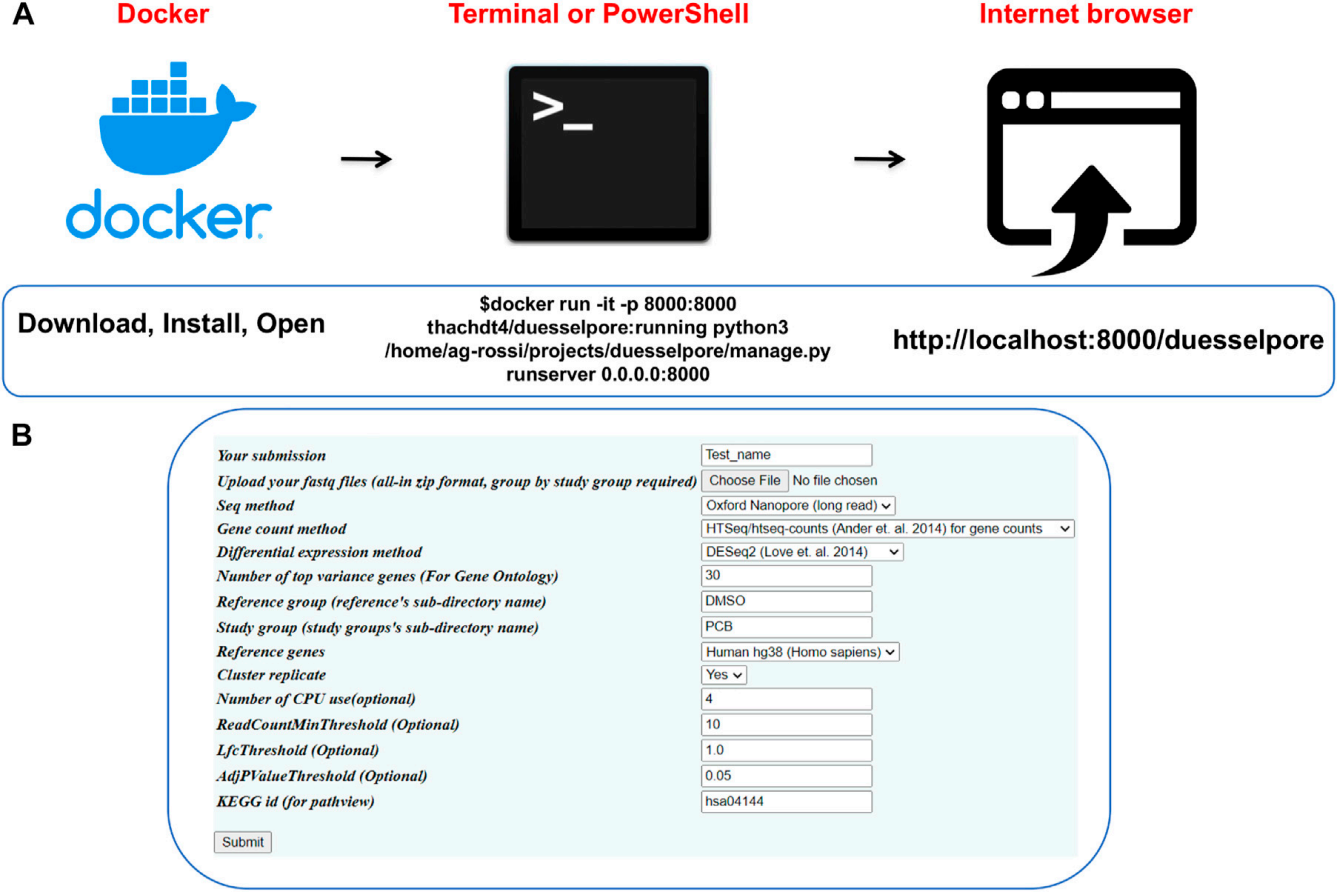
Operating Duesselpore. **(A)** Depicted are the required steps to launch Duesselpore^TM^ on Linux/MacOS (Terminal) or Windows (PowerShell). **(B)** The interface can be accessed *via* the user’s browser.

As input, Duesselpore^TM^ takes multiple FASTQ files that are the output of base-called ONT or Illumina data. The output includes a quality report, gene counts and expression, and gene ontology and disease pathway optionally. Duesselpore^TM^ can be installed in three steps (Figure 2A), and the user interface (Figure 2B) allows the user to fine-tune the sequencing analysis settings based on the experimental design of choice.

To validate Duesselpore^TM^ *,* immortalized HaCaT keratinocytes were stimulated with 3,3′,4,4′,5-pentachlorobiphenyl (PCB126) or solvent treated with 0.1% DMSO for 24 h. PCB126 was chosen as the mode of action because it is well understood how it regulates gene expression. According to their lipophilic properties, PCB126 molecules can easily pass through the cell membrane and bind the aryl hydrocarbon receptor (AHR), a cytosolic transcription factor trapped in a multiprotein complex. Upon ligand binding, the multiprotein complex dissociates, and the AHR translocates into the nucleus, where it dimerizes with the aryl hydrocarbon receptor nuclear translocator (ARNT) and binds to xenobiotic responsive elements (XRE) in the genome. This signaling pathway induces the expression of various genes encoding for Phase I and Phase II biotransformation enzymes (Bock and Köhle, 2006). Amongst these genes are the members of the superfamily cytochrome P450 (CYP) *CYP1A1*, *CYP1B1*, and aldehyde dehydrogenases (ALDH) *ALDH3A1* as well as genes encoding for proteins involved in cell proliferation, differentiation, and apoptosis (Vogeley et al., 2019).

Total RNA was isolated and transcribed into complementary DNA (cDNA) as described. cDNAs were barcoded, loaded into the same flowcell, and sequenced over a period of 72 h to ensure a sufficient number of reads for further downstream analyses. The raw FAST5 output files were base-called with Guppy, ONTs base-calling algorithm, and demultiplexed (barcode sorted). Base-calling and barcode demultiplexing were not included in Duesselpore^TM^ because these steps can be performed directly by the sequencer software (e.g., MinKNOW for ONT or Local Run Manager for Illumina). The generated FASTQ files were then concatenated and analyzed with Duesselpore^TM^.

Duesselpore^TM^ provides a mapping summary, including the number of reads, the number of reads mapped, and the percentage of mapped reads (Table 1). Secondary mappings were excluded as those are not considered when these sequences are assigned to genetic features. For clarification, each replicate represents a different passage of cells analyzed on a different flow cell.

**TABLE 1:** Mapping summary of sequences aligned with minimap2.

	PCB_replicate1	PCB_replicate2	PCB_replicate3	DMSO_replicate1	DMSO_replicate2	DMSO_replicate3
*nreads*	4929644	1561372	3624589	2877111	2122355	4027410
*Read mappings*	4995752	1586505	3651019	2918088	2150179	4055402
*Secondary*	0	0	0	0	0	0
*Supplementary*	66108	25133	26430	40977	27824	27992
*Duplicates*	0	0	0	0	0	0
*%mappings*	87.66	92.94	75.96	92.93	89.881	78.79

**FIGURE 3:**
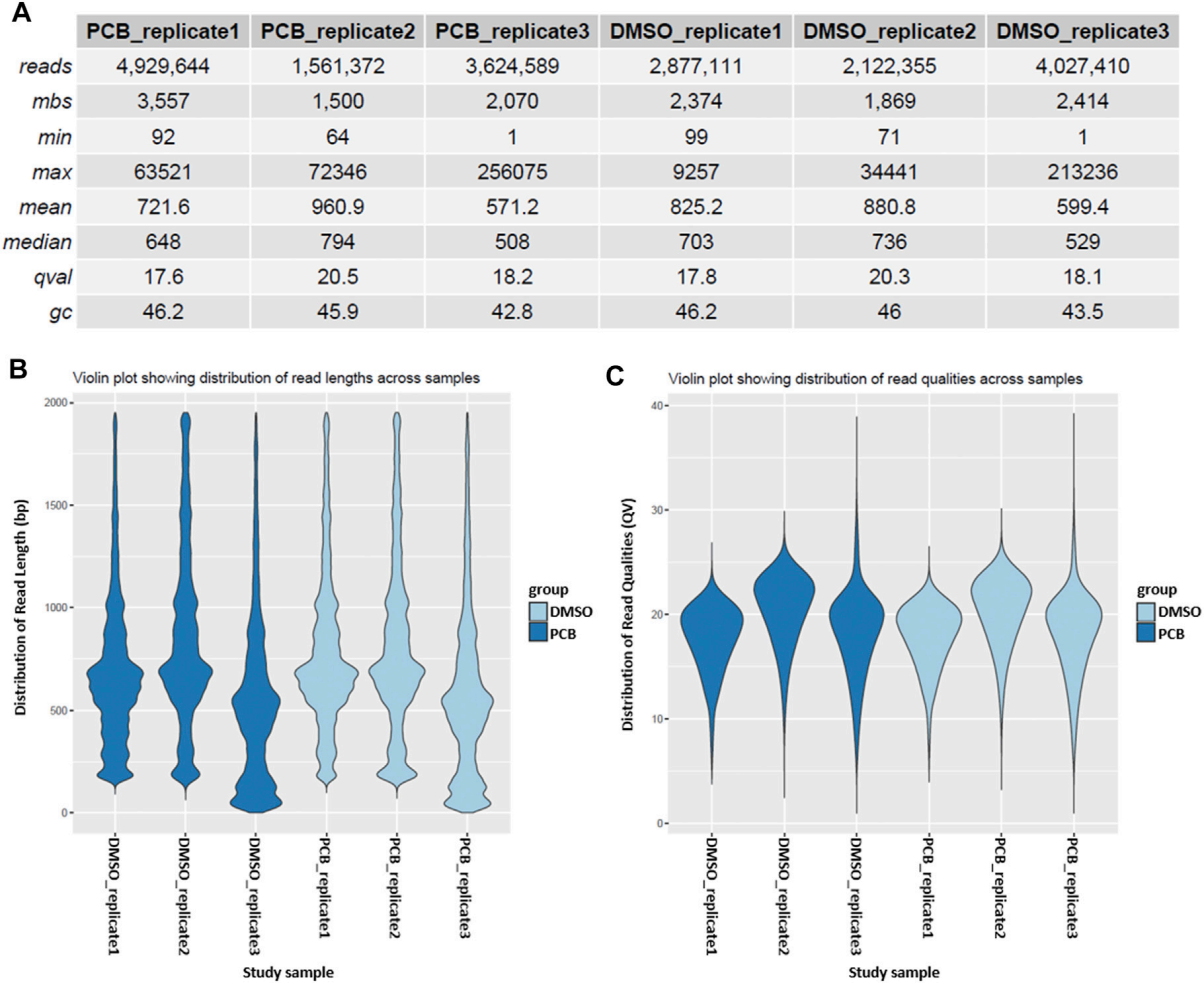
Sample summary and quality control. **(A)** Summary of the reads of every sample. The table provides information about the number of reads, million base sequences (**mbs**), minimal and maximum read length, mean and median read length, quality score (**qval**), and GC content (**gc**). Violin plots depict the distribution of read length **(B)** and the distribution of read quality **(C)** across different samples.

A more detailed sample overview is provided in the sample summary table, which contains information about the read length, million base sequences (mbs), mean quality score (qval), and GC content (gc) (Figure 3A). Additionally, read length and quality score distributions are depicted in violin plots (Figures 3B,C).

**FIGURE 4:**
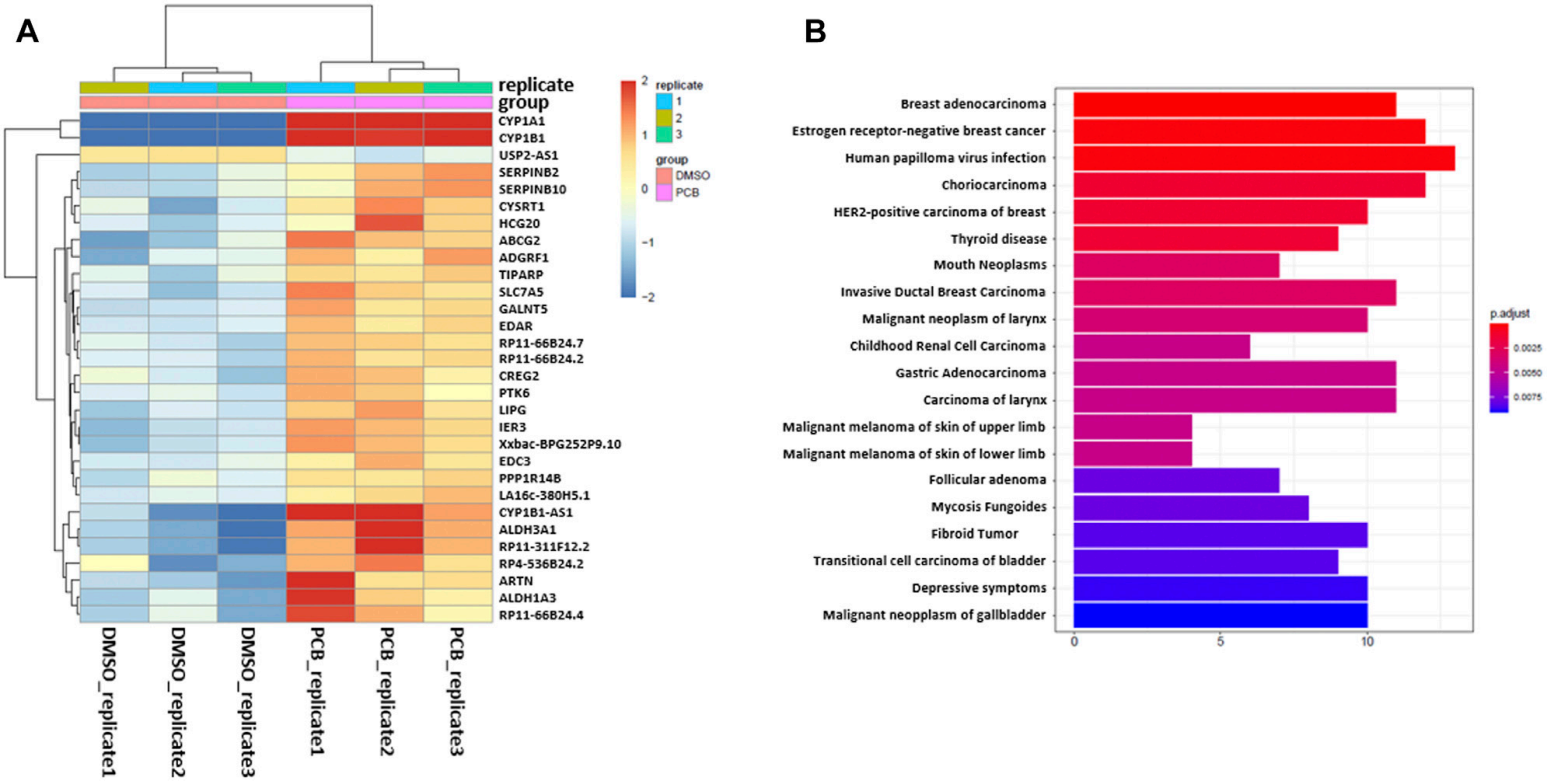
Variance of gene expression and enrichment analysis. **(A)** Heatmap showing the top 30 genes with the highest variance after normalizing for the CPM. **(B)** Enrichment analysis based on the DisGeNET database, revealing diseases, which are associated with the differentially expressed genes. X-axis shows the number of genes involved in each disease.

After carrying out sample quality control, Duesselpore^TM^ performs differential gene expression (DGE) analysis. Therefore, the user is able to choose between the packages *DESeq2* or *edgeR.* Both packages use statistical models based on the negative binomial distribution and are the most commonly used tools. It is recommended to use *DESeq2* when the number of replicas is relatively low (below 5), as this tool shows the highest consistency in the identification of significantly differentially expressed (SDE) genes and shows the lowest false discovery rate (Seyednasrollah et al., 2015). The results of the differentially expressed gene (DEG) analysis are provided as an excel file, and in our data set, the genes *CYP1A1*, *CYP1B1*, *ALDH3A1* as well as other established AHR target genes, such as *ATP-binding cassette super-family G member 2* (*ABCG2*), *plasminogen activator inhibitor-2* (*SERPINB2*), and *TCDD-inducible poly [ADP-ribose] polymerase* (*TiPARP*), are among the SDE genes. The regulation of *CYP1A1*, *CYP1B1*, and *ALDH3A1* was confirmed by qRT-PCR (Supplementary Figures S1A–C). The top 30 genes with the highest variance are depicted in a variance heatmap (Figure 4A), also provided by Duesselpore^TM^. For this purpose, the counts per gene were normalized to the counts per million (CPM) scaling factor (Robinson et al., 2009). The number of depicted genes can be defined in the user interface under the menu item “Number of top variance genes.”

Besides the detection of differentially expressed genes, Duesselpore^TM^ will also conduct gene set enrichment analyses (GSEA), enrichment analysis based on the DisGeNET (Piñero et al., 2015), and pathway-based data integration and visualization focusing on KEGG (Kyoto Encyclopedia of Genes and Genomes) pathways (Luo and Brouwer, 2013) (Supplementary Figure S2). These features will help the user to put the data into context and to identify potential signaling pathways and disease associations based on the SDE genes. To select a certain KEGG pathway, the user needs to provide a KEGG identifier and enter it into the webform under the menu item “KEGG id (for pathview).” PCB126 is highly toxic and exhibits mutagenic potential, which is not solely based on its capability to activate the AHR (Lauby-Secretan et al., 2013). Hence, genes regulated as a consequence of PCB126 exposure were associated with various malign neoplasias according to the DisGeNET database (Figure 4B). The relationship between genes and disease is depicted in a network plot either in a non-circular (Supplementary Figure S2A) or circular (Supplementary Figure S2B) format.

Duesselpore^TM^ outputs were successfully validated by using other datasets (data not shown). Here, the ability to detect PCB126-induced gene expression proves the functionality of Duesselpore^TM^ *.*

## Discussion

Over the last decade, RNA-sequencing methods have become more affordable and advanced to a commonly used technique to analyze genome-wide transcriptomes. While each step of sequencing has been drastically simplified, i.e., sample preparation due to the implementation of preparation kits, users are still struck by the size and the amount of raw data that require further analysis. This type of analysis often needs bioinformatics expertise and a certain amount of computational resources. In order to reduce this burden, we developed Duesselpore^TM^, a local web server that delivers a solution without interfering with the mobile characteristics of ONT sequencers, including the absence of an internet connection. Duesselpore^TM^ functionality was tested using ONT reads obtained by DMSO or PCB126 treated HaCaT keratinocyte samples. Among the DEGs, many prototypic genes that are regulated by the AHR, such as *CYP1A1*, *CYP1B1*, *ALDH3A1*, or *SERPINB2*, were upregulated in the test data set. The gene set enrichment analysis revealed an upregulation of gene patterns that are connected with different malign neoplasias. This observation is associated with the mutagenic potential of PCB126 and the fact that AHR activity is enhanced in many different tumor entities (Murray et al., 2014). These results were expected and thus validate the data obtained by Dusselpore^TM^ analysis (Bock and Köhle, 2006; Lauby-Secretan et al., 2013).

Duesselpore^TM^ interface allows the selection of five commonly used genomes (human, rat, mouse, zebrafish, and *C. elegans*), provides different pipelines to analyze RNA-Seq data, and by using publicly available tools, it helps users to perform advanced bioinformatical data analysis and generates figures and tables that are suitable for publication. Most of these tools require various dependencies, which is the reason why we encapsulated and compiled this workflow into a Docker image, making Duesselpore^TM^ independent from the host’s machine. All these properties grant the user strong flexibility in analyzing data even without profound bioinformatic knowledge. Furthermore, Duesselpore^TM^ is not limited to the analysis of ONT-derived data, but it also allows the analysis of Illumina and PacBio data (Supplementary Material). Moreover, Duesselpore^TM^ runs locally on the user’s machine, thus, data are not uploaded to the server. This is particularly helpful in avoiding risks associated with General Data Protection Regulation violations, security, and privacy.

In summary, we developed and successfully validated Duesselpore^TM^ as a web tool, which is able to analyze in detail ONT and NGS (PacBio or Illumina) derived RNA-Seq data without the requirement of a profound bioinformatical background. This will further democratize the analysis of NGS- and ONT-derived transcriptome sequencing data.

## Declarations

Code availability and data: please read Duesselpore manual for more information and software links:

https://github.com/thachnguyen/duesselpore.

Docker image:

https://hub.docker.com/repository/docker/thachdt4/duesselpore:running.

Test dataset (DMSO PCB standard and lightweight):

• https://iufduesseldorf-my.sharepoint.com/:u:/g/personal/thach_nguyen_iuf-duesseldorf_de/EWIk4CLauThHk61_5rItjEcBrSUl1a3_oZ6QxjfdDmdqsA?e=kRPvaa
• https://iufduesseldorf-my.sharepoint.com/:u:/g/personal/thach_nguyen_iuf-duesseldorf_de/ES4BsdfJSKNHl-mDUR3BogcB1HpDzV7eZ1kTikAd2Esq8w?e=lfDF9E

Sample result from ONT:

https://github.com/thachnguyen/duesselpore/blob/main/sample_result/nanopore/sample_result_ONT.zip

Sample result from Illumina:

https://github.com/thachnguyen/duesselpore/raw/main/sample_result/illumina/test_illumina.zip.

Sample result from PacBio:

https://github.com/thachnguyen/duesselpore/blob/main/sample_result/pacbio/Test_Pac_bio.zip.

## Data Availability

The datasets presented in this study can be found in online repositories. The names of the repository/repositories and accession number(s) can be found in the article/Supplementary Material.
