# Supplementary figures and images for: Rapid and simple analysis of short and long sequencing reads using DuesselporeTM
Source: Front Genet. 2022 Aug 11;13:931996. doi: 10.3389/fgene.2022.931996 (PMC9403543; doi:10.3389/fgene.2022.931996)

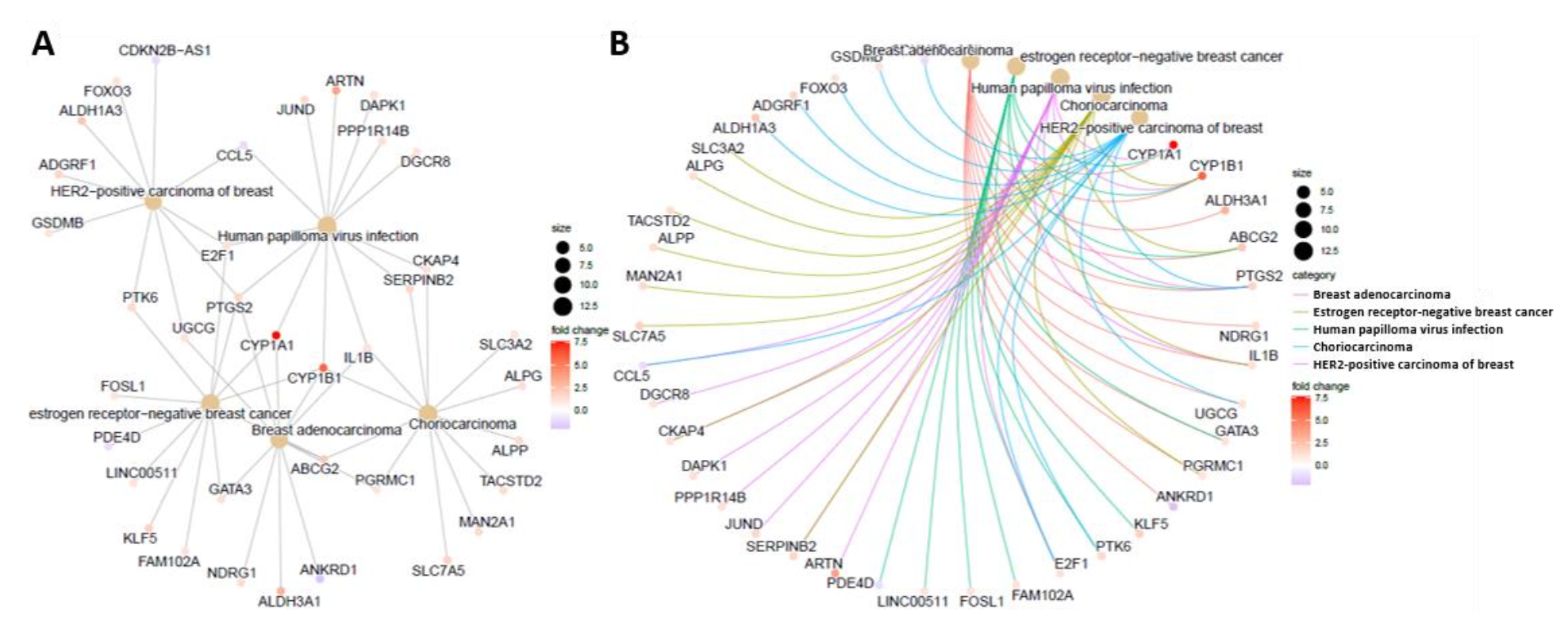

Supplement: Supplementary file 1 [file Image2.PNG]

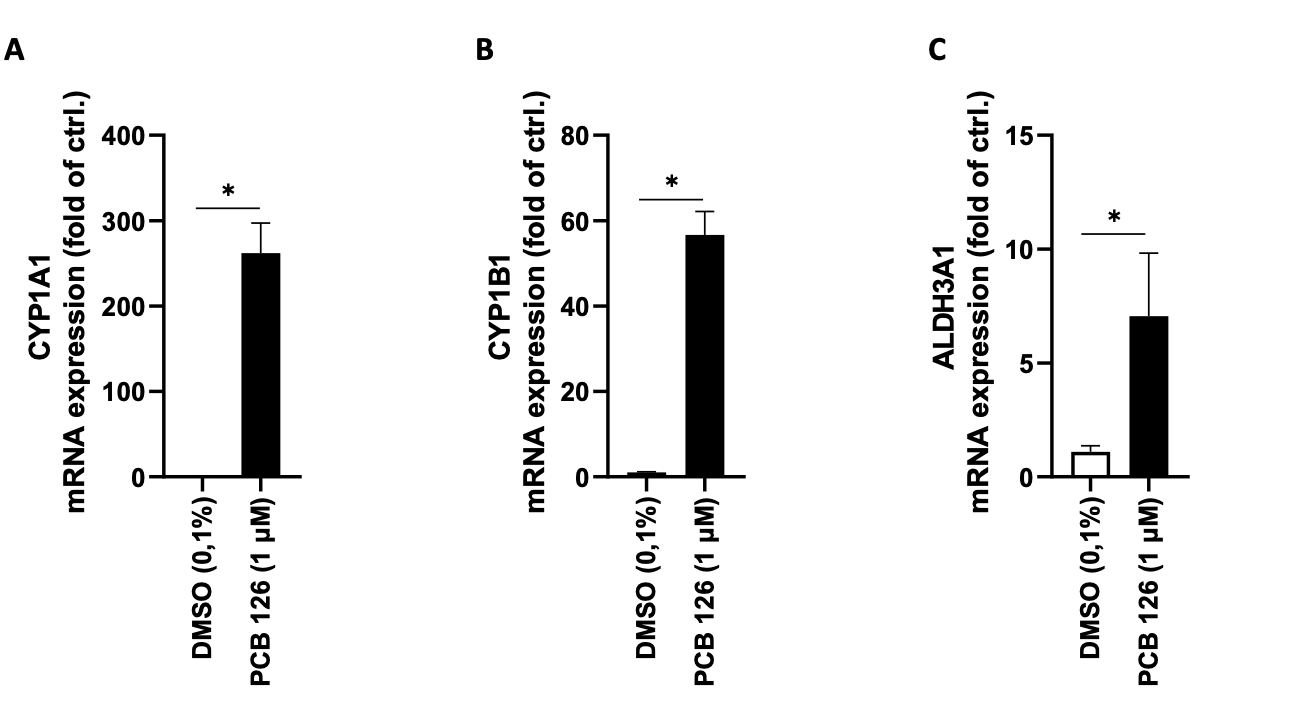

Supplement: Supplementary file 2 [file Image1.PNG]
