# Supplementary material for: Rapid and simple analysis of short and long sequencing reads using DuesselporeTM
Source: Front Genet. 2022 Aug 11;13:931996. doi: 10.3389/fgene.2022.931996 (PMC9403543; doi:10.3389/fgene.2022.931996)
Supplement: Supplementary file 3 [file DataSheet1.docx]

# Duesselpore^TM^ web server manual

## 1. Install and configure Duesselpore^TM^

### 1.1. System requirement

* CPU: 2.0 GHz 4 cores or higher
* System memory (RAM): 4 GB or higher
* Hard disk: 100 GB free space
* Host operating system Windows 10, Linux, or MacOS (64-bit)

16 GB memory is recommended to make Duesselpore^TM^ analysis faster. The hard disk free space should be at least three times the size of your experimental dataset.

Test data were analyzed using a E15 (11th Gen Intel® Core™ i7-1165G7 @ 2.80GHz × 8, 16 GB Memory, 500 GB SSD) laptop.

### 1.2. Installation

#### 1.2.1 Web server setup

Fast setup:

1. Download and install Docker from <https://www.docker.com/>
2. Open Windows Power Shell on Windows or Terminal on Linux or MacOS and type: $docker run -it -p 8000:8000 thachdt4/duesselpore:running python3 /home/ag-rossi/projects/duesselpore/manage.py runserver 0.0.0.0:8000
3. Open your browser and type <http://localhost:8000/duesselpore>

Step-by-step setup:

Download and install Docker from <https://www.docker.com/>.

On Linux or Mac OS, depending on your computer configuration, the swap memory might need to be increased (or virtual memory volume to 20GB). This step is not required on Windows 10 as it uses elastic virtual memory.

After installation, open the Windows Power Shell on Windows (Terminal on Linux or MacOS), Duesselpore^TM^ can be activated by a single command. You may have to use superuser (or admin/root) for the following command (on Windows, please make sure that Docker has already started, see screenshot below):


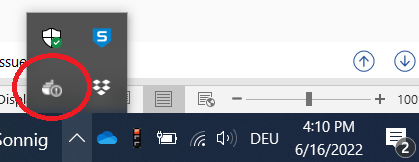


$docker run -it -p 8000:8000 thachdt4/duesselpore:running python3 /home/ag-rossi/projects/duesselpore/manage.py runserver 0.0.0.0:8000

If you see the messages

“Django version 3.2.8, using settings 'NGS_webserver.settings'

Starting development server at http://0.0.0.0:8000/

Quit the server with CONTROL-C.”

The server will successfully start.


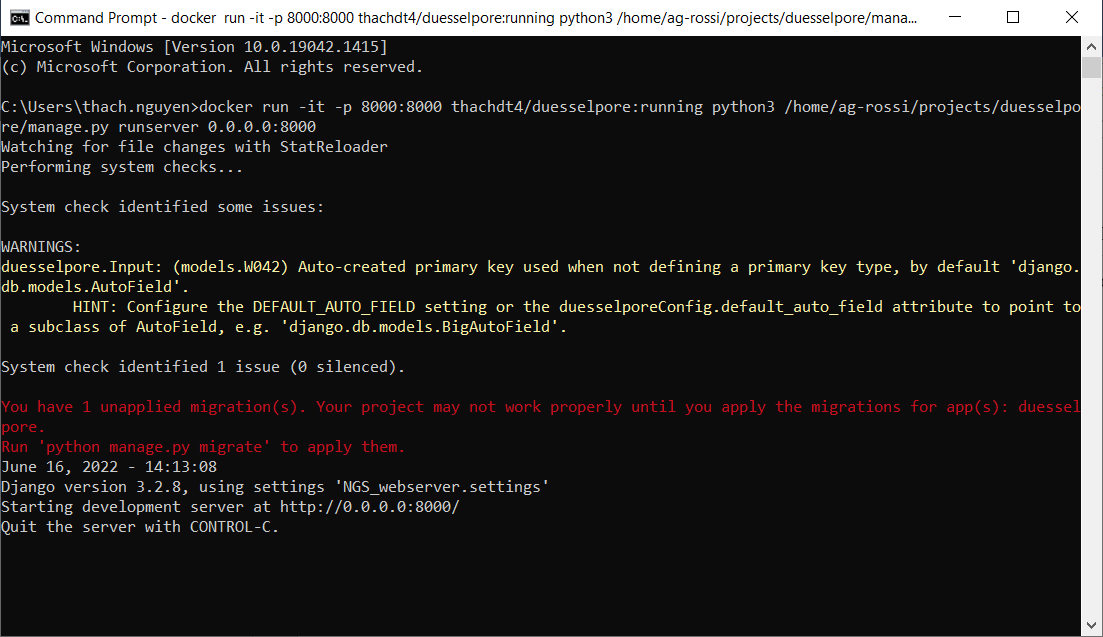


#### A user account can be added to the docker group to use Docker without sudo commands

$ sudo usermod -aG docker $USER

$ sudo chmod 666 /var/run/docker.sock

1.2.2. Test data

Download two test datasets:

- Full test data (14.6 GB): https://iufduesseldorf-my.sharepoint.com/:u:/g/personal/thach_nguyen_iuf-duesseldorf_de/EWIk4CLauThHk61_5rItjEcBOP4CJstbyCN9yN3ty36A7g?e=zRUf1T
- Lightweight test data (2.1 GB): https://iufduesseldorf-my.sharepoint.com/:u:/g/personal/thach_nguyen_iuf-duesseldorf_de/ES4BsdfJSKNHl-mDUR3BogcBEmdOawVTRy-eRXU3-XeG2A?e=Kq9O2e

### 2.2. Using the web server

#### 2.2.1. Access the web server

After activating the web server, Duesselpore^TM^ can be used within your Local Area Network (LAN) with a regular web browser (e.g., Firefox or Google Chrome port: 8000). Please note, an internet connection is not required.

<http://localhost:8000/duesselpore>

The web server can access via alternative ways 1) your Docker container network interface (default 172.17.0.2); 2) on the Docker container (address: localhost)

#### 2.2.2. Data preparation

Users can upload FASTQ files as one compressed zip file. Each subfolder contains several replicas with one experimental condition.

NOTE: files and folders’ names must contain only alphabetic and numeric characters. Below is an example of data separated into two conditions, ‘condition1’ and ‘condition2’. Please check the structure and the directory name of your data carefully, all the name of the analysis are generated by directory and file names.

fastq/(folder)
├── condition1 (subfolder)
│ ├── condition1_replica1.fastq (single fastq file)
│ └── condition1_replica2.fastq (single fastq file)
└── condition2 (subfolder)
 ├── condition2_replica1.fastq (single fastq file)
 ├── condition2_replica2.fastq (single fastq file)
 └── condition2_replica3.fastq (single fastq file)

If multiple FASTQ files need to be concatenated into a single file, please use the command below:

On Linux and Mac OS terminal:

$ cat /path/to/fastq/files/*.fastq > /your/new/location/output.fastq

On Windows command prompt (NOTE: path symbol is different):

$ type \path\to\fastq\files\*.fastq> \your\new\location\output.fastq

#### 2.2.3. Setup running parameter:

Most user interface settings are already tuned to analyze ONT data. However, the users will have the option to change these settings based on the experimental design and the sequencing methods used.

First, choose one group amongst the others to select your reference group. Select the gene (transcriptome) and the counting method, then select the differential expression algorithm of your choice. Other parameters can be customized (e.g., top variance, ReadCountMinThreshold, Logfold change threshold, adjPValueThreshold). Users will also be able to perform gene ontology and disease pathways analysis by providing the id number that can be found here: https://www.genome.jp/kegg/pathway.html

This analysis is, however, tied to an internet connection.

After submission, the user can wait for the result (please note that the browser will need to be kept open).

In case the web browser accidentally closed/crashed, user can login into the Docker and get the result at two directories:

/home/ag-rossi/projects/duesselpore/duesselpore/users_file

/home/ag-rossi/projects/duesselpore/duesselpore/static/results

Advanced users can customize the RNA.R code to develop a new workflow.

The figure below shows the web input form.


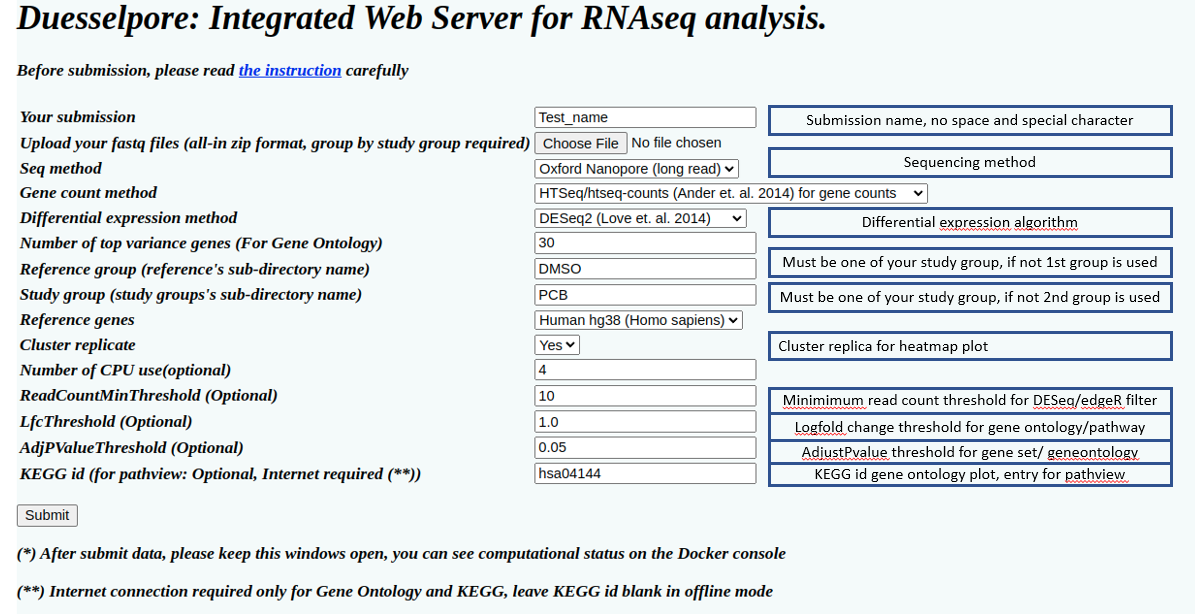


#### 2.2.4. Collecting the results:

The analysis run time depends on the dataset size and the system specifications. For our standard dataset, which contains six replicates, and approximately 16 million reads (around 15 Gb), the run time is around 6 hours. For the lightweight test data, the running time is approximately 1 hour.

When the analsyis is completed, the users will be able to download the results from the browser. The interactive HTML file is exported with different plots. Users can continue the offline analysis on the Docker container directory at /home/ag-rossi/duesselpore/users_file/{your session id}. Experienced users will also be able to further analyze their data by editing the R script. Since NGS data tend to take up hard disk space, we recommend erasing the Docker container's data regularly or recreating a new running container from our Docker image. The sample result can be found in the Supporting Information or sample_result/report.html in sample_result.zip.

While most analyses do not require an internet connection, gene ontology and disease pathways (optional function) will need an internet connection.
